# Expression and functional significance of phosphoenolpyruvate carboxykinase 1 in uveal melanoma

**DOI:** 10.1038/s41420-024-01963-y

**Published:** 2024-04-26

**Authors:** Hui-e Bi, Jie Zhang, Yujia Yao, Suyu Wang, Jin Yao, Zhijiang Shao, Qin Jiang

**Affiliations:** 1 The Affiliated Eye Hospital, The Fourth School of Clinical Medicine, Nanjing Medical University, Nanjing, China; 2 Department of Ophthalmology, The Affiliated Suzhou Hospital of Nanjing Medical University, Suzhou, China; 3 Obstetrics and Gynecology Department, The Affiliated Zhangjiagang Hospital of Soochow University, Suzhou, China

**Keywords:** Oncogenes, Eye cancer

## Abstract

Uveal melanoma (UVM), an uncommon yet potentially life-threatening ocular cancer, arises from melanocytes in the uveal tract of the eye. The exploration of novel oncotargets for UVM is of paramount importance. In this study, we show that PCK1 (phosphoenolpyruvate carboxykinase 1) expression is upregulated in various UVM tissues as well as in primary UVM cells and immortalized lines. Furthermore, bioinformatics studies reveal that PCK1 overexpression in UVM correlates with advanced disease stages and poor patient survival. Genetic silencing (utilizing viral shRNA) or knockout (via CRISPR/Cas9) of PCK1 significantly curtailed cell viability, proliferation, cell cycle progression, and motility, while provoking apoptosis in primary and immortalized UVM cells. Conversely, ectopic overexpression of PCK1, achieved through a viral construct, bolstered UVM cell proliferation and migration. Gαi3 expression and Akt phosphorylation were reduced following PCK1 silencing or knockout, but increased after PCK1 overexpression in UVM cells. Restoring Akt phosphorylation through a constitutively active mutant Akt1 (S473D) ameliorated the growth inhibition, migration suppression, and apoptosis induced by PCK1 silencing in UVM cells. Additionally, ectopic expression of Gαi3 restored Akt activation and counteracted the anti-UVM cell effects by PCK1 silencing. In vivo, the growth of subcutaneous xenografts of primary human UVM cells was significantly inhibited following intratumoral injection of adeno-associated virus (aav) expressing PCK1 shRNA. PCK1 depletion, Gαi3 downregulation, Akt inhibition, proliferation arrest, and apoptosis were detected in PCK1-silenced UVM xenografts. Collectively, our findings demonstrate that PCK1 promotes UVM cell growth possibly by modulating the Gαi3-Akt signaling pathway.

## Introduction

Uveal melanoma (UVM) is a rare and aggressive eye cancer originating from melanocytes in the uveal tract, posing a significant threat to vision and life [1–4]. In Europe and USA, the yearly occurrence rate stands at approximately six cases per million individuals, it is yet the most common primary intraocular malignancy in adults [2, 5, 6]. Risk factors include genetic predisposition, ultraviolent (UV) radiation, congenital ocular melanocytosis and ocular melanocytoma, and diagnosis relies on comprehensive eye examinations and imaging techniques including ultrasound and optical coherence tomography (OCT) [1–4]. The prognosis can be poor, as metastasis, often to the liver, is associated with limited treatment options [2, 4, 7]. The management of UVM is contingent upon the tumor’s size and location [1–3]. Strategies for the localized tumors include surgical resection, radiation therapy, and thermotherapy [1, 3–5, 8]. In specific cases, enucleation may be a required intervention [1, 3–5, 8]. Ongoing research is focusing on the potential of targeted therapies as treatment avenues for advanced UVM [1, 3–5, 8].

Targeted therapies for UVM encompass a range of approaches designed to suppress specific signaling pathways or genetic mutations that drive the progression of cancer [3–5, 8]. These strategies include the use of c-KIT inhibitors [9], BRAF/MEK inhibitors [10], PI3K-Akt inhibitors [11], and also immune checkpoint inhibitors [12]. These targeted therapies are selected based on the genetic and molecular characteristics of UVM [1–4]. While the effectiveness of targeted therapies may vary, ongoing research and clinical trials continue to expand novel therapeutic targets and corresponding treatments available for this rare and challenging form of eye cancer [3–5, 8].

Phosphoenolpyruvate carboxykinase 1 (PCK1) is a key enzyme in gluconeogenesis, the metabolic pathway responsible for the synthesis of glucose from non-carbohydrate precursors [13–15]. PCK1 plays a crucial role in regulating blood glucose levels, as it catalyzes the conversion of oxaloacetate to phosphoenolpyruvate [13–15]. It is a key step in the production of glucose from substrates including lactate, glycerol, and amino acids [13–15]. PCK1 is primarily found in the liver and kidney and is subject to intricate regulation by various hormonal signals, including insulin and glucagon, to maintain glucose homeostasis [16, 17]. Dysregulation of PCK1 activity can contribute to metabolic disorders such as diabetes and may have implications for understanding glucose metabolism and metabolic diseases [16, 17]. Recent research findings have documented elevated expression levels of PCK1 (or PCK2) in various cancers, such as colon cancer, pancreatic cancer, lung cancer, melanoma, lymphoma, and metastatic breast cancer cells [18–21]. These observations suggest that PCK1 may serve a non-gluconeogenic role in the regulation of tumor development. Nevertheless, the expression and functional significant of PCK1 in UVM have not been thoroughly studied.

## Results

### PCK1 overexpression in uveal melanoma correlates with poor overall survival and advanced disease stage

**Fig. 1:**
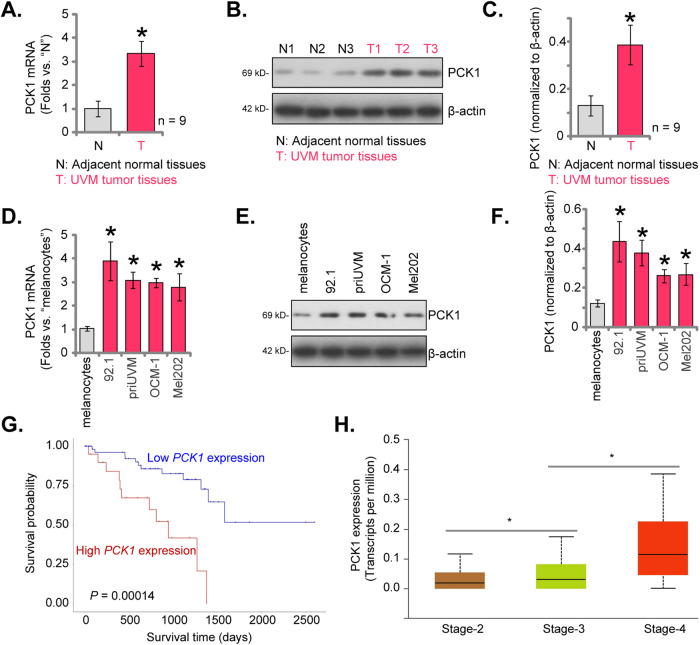
PCK1 overexpression in uveal melanoma correlates with poor overall survival and advanced disease stage. Expression of *PCK1* mRNA and protein in described UVM tissues (“T”) and surrounding normal tissues (“N”) was tested by qRT-PCR and Western blotting assays (**A**–**C**, *n* = 9 tissues per group). Expression of *PCK1* mRNA (**D**) and protein (**E** and **F**) in described UVM cells and primary human melanocytes (“melanocytes”) was tested as well (*n* = 5, biological repeats). TCGA Kaplan-Meier survival analyses demonstrated the association between *PCK1* expression and overall survival in UVM patients in TCGA database (**G**). TCGA cohort demonstrates *PCK1* expression in UVM patients with different pathological stages (**H**). *P < 0.05 vs. “N” tissues or– “melanocytes” (**A**–**F**). *P < 0.05 (**H**).

We first examined the expression profile of PCK1 in UVM tissues. Our analyses enrolled nine primary UVM patients (*n* = 9) and primarily focused on comparing PCK1 expression between UVM tissues (“T”) and adjacent normal tissues (“N”). A remarkable increase in *PCK1* mRNA expression in UVM tissues was detected, as compared to the matched normal tissues (Fig. 1A). Western blotting analyses demonstrated a significant elevation in PCK1 protein levels within UVM tissues from three representative patients (Fig. 1B). A thorough examination of PCK1 protein blotting data across all nine tissue sets confirmed a significant upregulation of PCK1 protein within UVM tissues (Fig. 1C).

The subsequent investigations aimed to evaluate PCK1 expression in various UVM cells, including primary human UVM cells (“priUVM”) and immortalized cell lines, including 92.1, OCM-1, and Mel202. A significant increase in *PCK1* mRNA expression in both primary and immortalized UVM cells was detected, in contrast to the primary human melanocytes (in Fig. 1D). Additionally, the upregulation of PCK1 protein was consistently observed in various UVM cells (Fig. 1E, F), while its expression remained substantially low in human melanocytes (Fig. 1E, F). Importantly, an analysis of The Cancer Genome Atlas (TCGA) database further reveals that PCK1 overexpression is associated with an unfavorable prognosis and poor survival in UVM patients (Fig. 1G). Additionally, overexpression of PCK1 is correlated with higher disease stage (Fig. 1H). Collectively, these results emphasize the robust upregulation of PCK1 in UVM, which is associated with poor overall survival and advanced disease stage, highlighting the potential significance of PCK1 in the progression of UVM.

### PCK1 silencing inhibits viability, proliferation, cell cycle progression and mobility of uveal melanoma cells

**Fig. 2:**
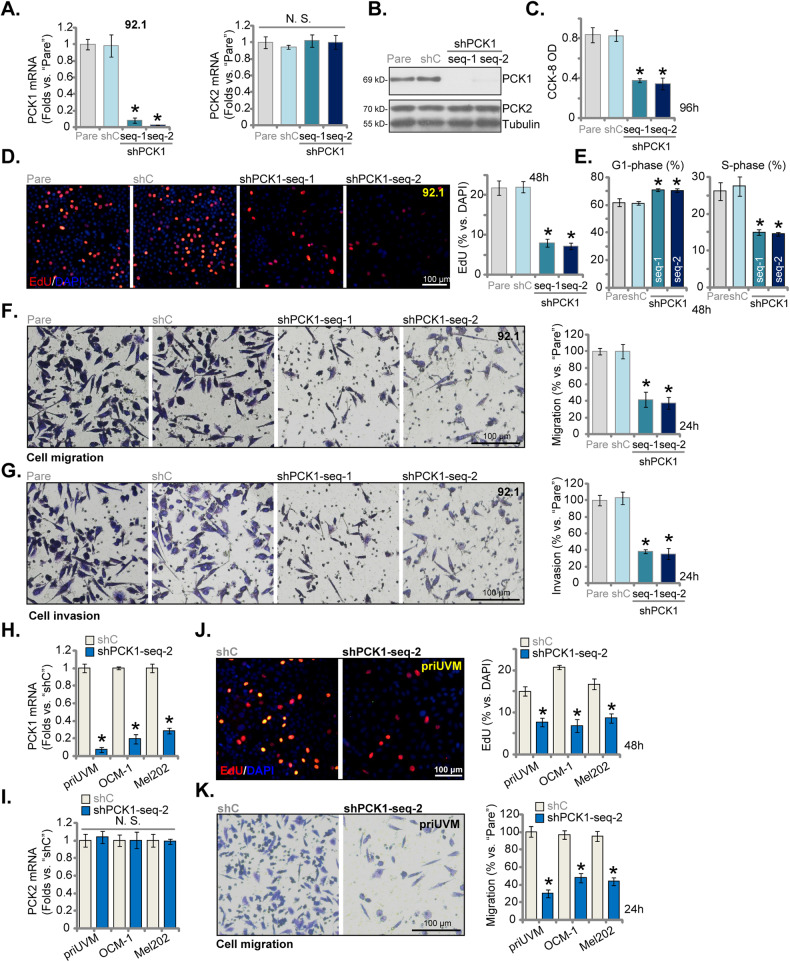
PCK1 silencing inhibits viability, proliferation, cell cycle progression and mobility of uveal melanoma cells. The stable 92.1 UVM cells with the lentiviral PCK1 shRNA (“shPCK1-seq-1” or “shPCK1-seq-2”, two different sequences) or the lentiviral control shRNA (“shC”) were established and PCK1 and PCK2 expression (both mRNA and protein) was examined (**A**, **B**). Subsequently, an equal number of the aforementioned 92.1 cells were cultured for specified durations, and cell viability, proliferation, cell cycle progression, in vitro cell migration, and invasion were tested via CCK-8 (**C**), EdU-nuclei staining (**D**) and PI-FACS analysis (**E**) as well as “Transwell” (**F**) and “Matrigel Transwell” (**G**) assays, respectively, with results quantified. The patient-derived primary human UVM cells, “priUVM,” and the immortalized lines (OCM-1 and Mel202), were engineered to stably express either “shPCK1-seq-2” or “shC”, mRNA expression of *PCK1* and *PCK2* was tested (**H**, **I**); An equal number of these UVM cells were further cultivated for designated hours, cell proliferation (**J**) and in vitro cell migration (**K**) were tested similarly. The numerical values are presented as the mean ± standard deviation (SD). “Pare” signifies the parental control cells. *Indicates statistical significance (*P* < 0.05) when compared to “Pare” or “shC” cells, while “N. S.” denotes a lack of statistical difference (*P* > 0.05). The experiments depicted in this figure were replicated five times (*n* = 5, biological repeats), consistently yielding similar results. The scale bar corresponds to 100 μm.

To investigate the potential role of PCK1 in UVM, we introduced lentivirus-packaged PCK1 shRNAs, namely “shPCK1-seq-1” and “shPCK1-seq-2” [22], into the established UVM cell line, 92.1. Stable cells were established following selection with puromycin. In comparison to 92.1 cells transfected with a scramble control shRNA, denoted as “shC,” we observed a significant reduction in both *PCK1* mRNA (Fig. 2A) and protein (Fig. 2B) expression in cells expressing shPCK1-seq-1 and shPCK1-seq-2. Importantly, the expression of PCK2 remained largely unchanged in 92.1 cells upon PCK1 silencing (Fig. 2A, B). Functional investigations further unveiled that the silencing of PCK1 by shRNA led to a decrease in the viability of 92.1 cells, as evidenced by a reduction in CCK-8 optical density (OD) values (Fig. 2C). Subsequently, the PI-FACS assay results revealed a noteworthy increase in the percentage of G1-phase 92.1 cells and a corresponding decrease in the percentage of cells in the S-phase upon silencing of PCK1 (Fig. 2E), supporting G1-S arrest induced by PCK1 shRNA (Fig. 2E). Furthermore, the application of PCK1 shRNAs had a significant impact on slowing down the in vitro migration and invasion of 92.1 cells, as evidenced by the results from the “Transwell” (Fig. 2F) and “Matrigel Transwell” (Fig. 2G) assays, respectively. In contrast, treatment with shC, as anticipated, had no significant effect on the viability (Fig. 2C), proliferation (Fig. 2D), cell cycle progression (Fig. 2E), in vitro migration (Fig. 2F), or invasion (Fig. 2G) of 92.1 UVM cells.

Next, experiments were carried out to investigate the impact of PCK1 silencing on other UVM cell types. We introduced lentivirus carrying shPCK1-seq-2 into patient-derived primary human UVM cells (“priUVM”) as well as other immortalized cell lines, OCM-1 and Mel202. Stable cell lines were once again established through puromycin-based selection. When compared to shC-expressing cells, *PCK1* mRNA expression was dramatically decreased in shPCK1-seq-2-experssing priUVM, OCM-1 and Mel202 cells (Fig. 2H), where *PCK2* mRNA was unchanged (Fig. 2I). In line with the effects observed in 92.1 cells, the silencing of PCK1 through shPCK1-seq-2 exhibited a consistent pattern of hindering proliferation in both primary and immortalized UVM cells, resulting in a significant reduction in the ratio of EdU-positive nuclei (Fig. 2J). Furthermore, the in vitro migration of UVM cells was also slowed upon treatment with shPCK1-seq-2 (Fig. 2K).

### PCK1 silencing induces apoptosis activation in uveal melanoma cells

**Fig. 3:**
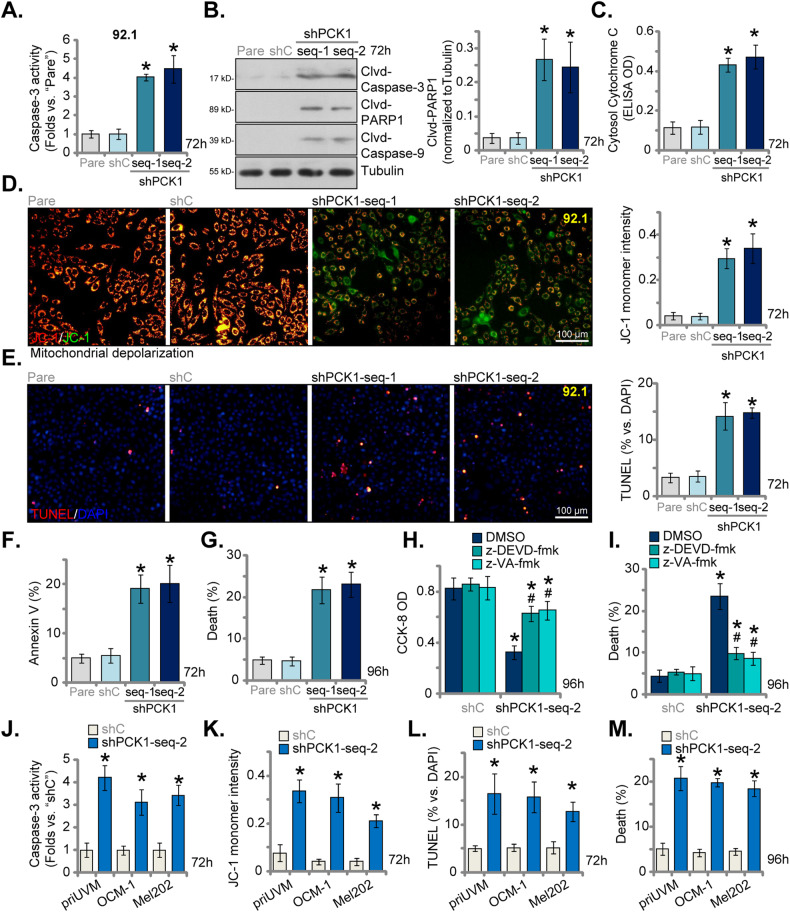
PCK1 silencing induces apoptosis activation in uveal melanoma cells. The stable 92.1 UVM cells with the lentiviral PCK1 shRNA (“shPCK1-seq-1” or “shPCK1-seq-2”, two different sequences) or the lentiviral control shRNA (“shC”) were established, and an equal number of the aforementioned 92.1 cells were cultured for specified durations. The cytosol lysates were obtained, the Caspase-3 activity (**A**), expression of listed apoptosis proteins (tested via Western blotting assays, **B**) and Cytochrome C content (tested via an ELISA kit, **C**) were measured. Mitochondrial depolarization was measured via JC-1 staining (**D**); Cell apoptosis was evaluated by nuclear TUNEL staining (**E**) and Annexin V FACS (**F**) assays, with cell death measured via Trypan blue staining assays (**G**). 92.1 UVM cells with”shPCK1-seq-2” or “shC” were treated with z-DEVD-fmk (40 μM), z-VAD-fmk (40 μM) or DMSO (0.1%) for designated hours, cell viability (CCK-8 assay, **G**) and death (**H**) were measured. The patient-derived primary human UVM cells, priUVM, and the immortalized lines (OCM-1 and Mel202) were engineered to stably express either “shPCK1-seq-2” or “shC”, and an equal number of the aforementioned UVM cells cultured for specified durations. The Caspase-3 activity (**J**), mitochondrial depolarization (tested via measuring JC-1 monomer intensity, **K**), cell apoptosis (measured by the nuclear TUNEL staining, **L**) and cell death (**M**) were tested similarly. The numerical values are presented as the mean ± standard deviation (SD). “Pare” signifies the parental control cells. *Indicates statistical significance (*P* < 0.05) when compared to “shC” cells. ^#^Indicates statistical significance (*P* < 0.05) when compared to “DMSO” treatment (**G** and **H**). The experiments depicted in this figure were replicated five times (*n* = 5, biological repeats), consistently yielding similar results. The scale bar corresponds to 100 μm.

Given the observed proliferation inhibition and cell cycle arrest in UVM cells upon PCK1 silencing, we studied whether apoptosis was induced as a consequence. PCK1 silencing via “shPCK1-seq-1” and “shPCK1-seq-2” led to an increase in Caspase-3 activity in 92.1 UVM cells (Fig. 3A). Moreover, levels of cleaved-Caspase-3, cleaved-Caspase-9, and cleaved-Poly(ADP-ribose) polymerase (PARP-1) were elevated in PCK1-silenced 92.1 cells (Fig. 3B). The content of cytosolic Cytochrome C, measured using an ELISA kit, was also increased following treatment with PCK1 shRNA in 92.1 cells (Fig. 3C). Significant mitochondrial depolarization was detected in shPCK1-expressing cells, as evidenced by the conversion of JC-1 red fluorescence (aggregates) to green fluorescence (monomers) (Fig. 3D). All these findings collectively support the activation of the mitochondrial apoptosis cascade in PCK1-silenced cells [23–25]. Importantly, the results unequivocally demonstrated that shPCK1-seq-1/2 treatment induced apoptosis in 92.1 UVM cells, as indicated by a significant increase in the TUNEL-positive nuclei ratio (Fig. 3E). Apoptosis was further substantiated by the elevated Annexin V ratio in shPCK1-expressing 92.1 cells (Fig. 3F). In contrast, treatment with shC failed to induce Caspase-PARP1-Cytochrome C activation (Fig. 3A–C), mitochondrial depolarization (Fig. 3D), and apoptosis (Fig. 3E, F) in 92.1 cells.

Substantial cell death was also evident in PCK1-silenced 92.1 UVM cells, as indicated by an increased number of Trypan blue-positive staining following shPCK1-seq-1/2 treatment (Fig. 3G). To assess the role of apoptosis in this cell death, we employed two well-established Caspase-apoptosis inhibitors: the Caspase-3 specific inhibitor z-DEVD-fmk and the pan Caspase inhibitor z-VAD-fmk. As depicted in Fig. 3H, the inhibitors largely mitigated the reduction in cell viability (CCK-8 OD) induced by shPCK1-seq-2 treatment (Fig. 3H) and reduced cell death (Fig. 3I) in 92.1 cells. These results provide strong support for apoptosis being the primary mechanism responsible for the cytotoxic effects of PCK1 silencing in 92.1 UVM cells.

In primary cells (priUVM) and other immortalized lines (OCM-1 and Mel202), PCK1 silencing through shPCK1-seq-2 (see Fig. 2) similarly led to increased Caspase-3 activity (Fig. 3J) and induced mitochondrial depolarization, as evidenced by the accumulation of JC-1 green monomers (Fig. 3K). Moreover, the elevated percentage of TUNEL-positive nuclei provided further evidence of apoptosis activation in shPCK1-seq-2-expressing UVM cells (Fig. 3L). Additionally, shPCK1-seq-2 triggered significant death in both primary and established UVM cells, resulting in an increased number of Trypan blue-stained cells (Fig. 3M).

### PCK1 knockout exerts significant anti-tumor effects in uveal melanoma cells

**Fig. 4:**
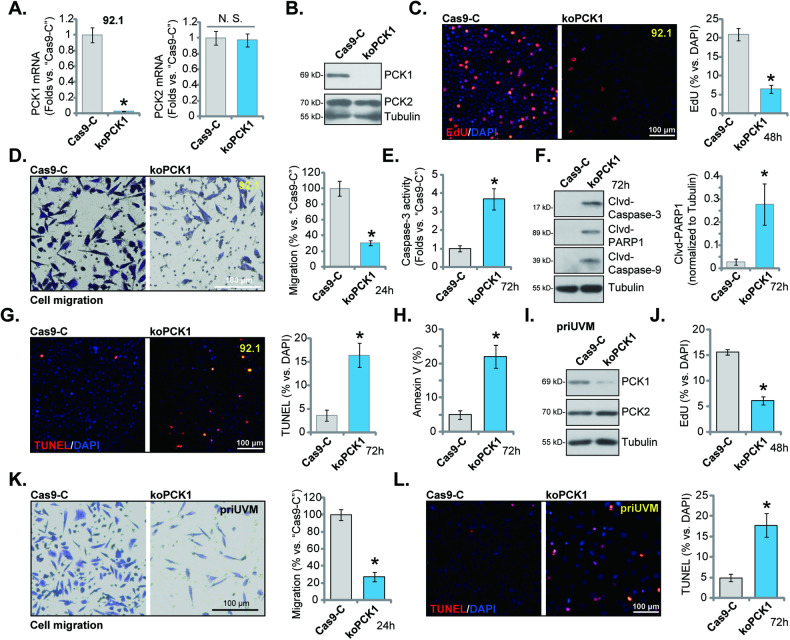
PCK1 knockout exerts significant anti-tumor effects in uveal melanoma cells. The single stable Cas9-expressing 92.1 cells with the lentiviral CRISPR/Cas9-PCK1-KO construct (“koPCK1”) or the CRISPR/Cas9-control construct (“Cas9-C”) were established, expression of PCK1/2 was measured (**A** and **B**). An equal number of the aforementioned 92.1 cells were cultured for specified durations, and cell proliferation and in vitro cell migration were tested via EdU-nuclei staining (**C**) and “Transwell” (**D**) assays, respectively. The cytosol lysates were obtained, the Caspase-3 activity (**E**) and expression of listed apoptosis proteins (via Western blotting assays, **F**) were measured. Cell apoptosis was tested by nuclear TUNEL staining (**G**) and Annexin V FACS (**H**) assays. The Cas9-expressing primary human UVM cells, priUVM, with the lentiviral CRISPR/Cas9-PCK1-KO construct (“koPCK1”) or the CRISPR/Cas9-control construct (“Cas9-C”) were established, and PCK1/2 protein expression examined (**I**). An equal number of the aforementioned priUVM cells was cultivated for designated hours, and cell proliferation, in vitro cell migration and apoptosis were tested via EdU-nuclei staining (**J**), “Transwell” (**K**) and TUNEL staining (**L**) assays, respectively. The numerical values are presented as the mean ± standard deviation (SD). *Indicates statistical significance (*P* < 0.05) when compared to “Cas9-C” cells. The experiments depicted in this figure were replicated five times (*n* = 5, biological repeats), consistently yielding similar results. The scale bar corresponds to 100 μm.

Subsequently, we employed the CRISPR/Cas9 strategy, as previously described [22], to generate stable single PCK1 knockout (KO) 92.1 UVM cells, or “koPCK1” cells. In comparison to control cells containing the CRISPR/Cas9 control construct (“Cas9-C”), the expression of *PCK1* mRNA (Fig. 4A) and protein (Fig. 4B) was markedly diminished in the koPCK1 92.1 cells, while PCK2 expression remained largely unchanged (Fig. 4A, B). The CRISPR/Cas9-induced PCK1 KO significantly impeded the proliferation of 92.1 cells, as indicated by a decrease in the percentage of EdU-positive nuclei (Fig. 4C). Furthermore, in vitro cell migration of 92.1 cells was notably reduced following PCK1 KO (Fig. 4D).Further analysis revealed an increase in Caspase-3 activity in koPCK1 92.1 cells (Fig. 4E), along with the induction of cleavage of Caspase-3, PARP1, and Caspase-9 (Fig. 4F), all indicative of apoptosis activation. Indeed, the TUNEL-positive nuclei ratio (Fig. 4G) and the percentage of Annexin V-positive stained cells (Fig. 4H) were both increased following PCK1 KO in 92.1 UVM cells. We applied the same CRISPR/Cas9 strategy to perform PCK1 KO in primary human UVM cells (priUVM), resulting in a significant depletion of PCK1 protein (“koPCK1”, Fig. 4I), while PCK2 protein expression remained unchanged (Fig. 4I). Consistent with the findings in 92.1 cells, PCK1 KO treatment hindered the proliferation (EdU incorporation) of priUVM cells (Fig. 4J) and reduced in vitro cell migration (Fig. 4K). Furthermore, PCK1 KO led to the activation of apoptosis, as evidenced by an increase in TUNEL-positive nuclei (Fig. 4L).

### PCK1 overexpression strengthens proliferation and migration of uveal melanoma cells

**Fig. 5:**
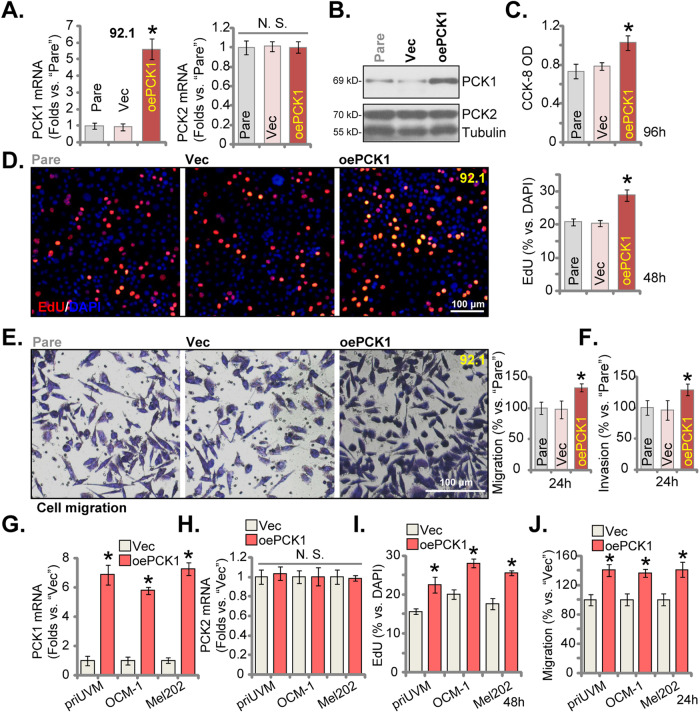
PCK1 overexpression strengthens proliferation and migration of uveal melanoma cells. The stable 92.1 cells with the lentivirus-packed PCK1-expressing construct (“oePCK1”) or the empty vector (“Vec”) were established and expression of PCK1/2 was measured (**A** and **B**). An equal number of the aforementioned 92.1 cells were cultured for specified durations, and cell viability, proliferation, in vitro cell migration and invasion were tested via CCK-8 (**C**), EdU-nuclei staining (**D**), “Transwell” (**E**) and “Matrigel Transwell” (**F**) assays, respectively. The patient-derived primary human UVM cells, priUVM, and the immortalized lines (OCM-1 and Mel202) were engineered to stably express either the lentivirus-packed PCK1-expressing construct (“oePCK1”) or the empty vector (“Vec”), and *PCK1/2* mRNA expression was tested (**G**, **H**). An equal number of the aforementioned UVM cells were cultured for specified durations, and proliferation and in vitro cell migration were tested via measuring EdU-nuclei ratio (**I**) and “Transwell” (**J**) assays, respectively, with results quantified. The numerical values are presented as the mean ± standard deviation (SD). “Pare” signifies the parental control cells. *Indicates statistical significance (*P* < 0.05) when compared to “Vec” cells. “N. S.” denotes a lack of statistical difference (*P* > 0.05). The experiments depicted in this figure were replicated five times (*n* = 5, biological repeats), consistently yielding similar results. The scale bar corresponds to 100 μm.

Based on the results above, we propose that further increasing PCK1 expression could exert tumor-promoting activity. Thus, the lentivirus-packed PCK1-expressing construct [22] was stably transduced to 92.1 UVM cells and stable cells, “oePCK1”, were formed after puromycin based selection. As compared to the control cells with the lentiviral vector (“Vec”), the mRNA (Fig. 5A) and protein (Fig. 5B) expression of PCK1 was dramatically increased in oePCK1 92.1 UVM cells. Whereas *PCK2* mRNA (Fig. 5A) and protein (Fig. 5B) expression was again unaltered. The functional studies showed that the viability, tested by CCK-8 OD, was increased in oePCK1 92.1 cells (Fig. 5C). In addition, ectopic overexpression of PCK1 promoted 92.1 cell proliferation and increased nuclear EdU incorporation (Fig. 5D). Furthermore, with PCK1 overexpression the in vitro cell migration (Fig. 5E) and invasion (Fig. 5F) were accelerated. The same lentiviral PCK1-expressing construct was also employed to establish PCK1-overexpressing cells (“oePCK1”) in the priUVM primary cells and other immortalized lines (OCM-1 and Mel202), causing substantial *PCK1* mRNA overexpression (Fig. 5G). Expression of *PCK2* mRNA was again unchanged (Fig. 5H). Importantly, in these oePCK1 UVM cells, cell proliferation (the ratio of EdU-stained nuclei, Fig. 5I) and in vitro cell migration (Fig. 5J) were also strengthened.

### PCK1 may play a significant role in Akt activation in uveal melanoma cells

**Fig. 6:**
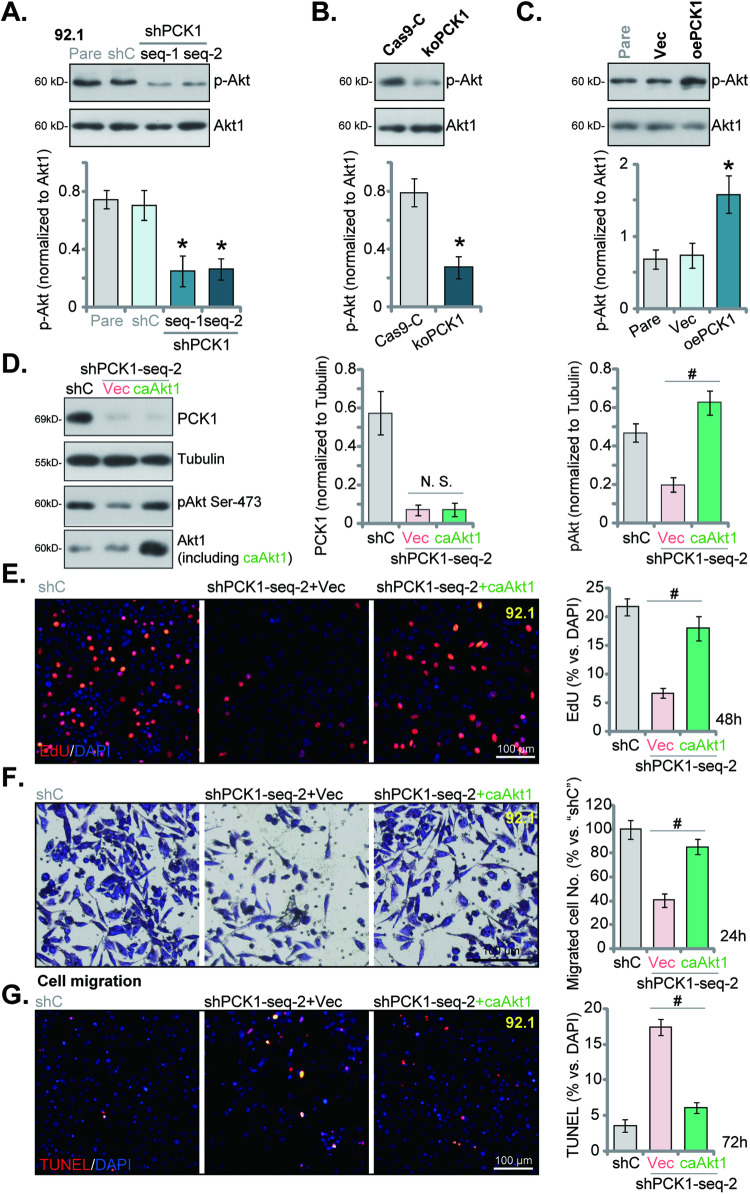
PCK1 may play a significant role in Akt activation in uveal melanoma cells. The 92.1 cells were engineered to stably express the lentiviral PCK1 shRNA (“shPCK1-seq-1” or “shPCK1-seq-2”, the lentiviral control shRNA (“shC”) (**A**), the lentiviral CRISPR/Cas9-PCK1-KO construct (“koPCK1”), the CRISPR/Cas9-control construct (“Cas9-C”) (**B**), the lentivirus-packed PCK1-expressing construct (“oePCK1”) or the empty vector (“Vec”) (**C**), p-Akt (at Ser-473) and Akt1 expression was tested. The shPCK1-seq-2-expressing 92.1 cells were subjected to stable transduction with a constitutively-active mutant Akt1 bearing the S473D mutation (“caAkt1”) or an empty vector (“Vec”). The expression levels of the specified proteins were shown (**D**). An equal number of the aforementioned 92.1 cells were cultured for specific durations, cell proliferation, migration, and apoptosis were assessed using EdU-nuclei staining (**E**), “Transwell” assays (**F**), and TUNEL-nuclei staining (**G**), respectively. The numerical values are presented as the mean ± standard deviation (SD). “Pare” signifies the parental control cells. *Indicates statistical significance (*P* < 0.05) when compared to “shC”/”Cas9-C”/”Vec” cells (**A**–**C**). ^#^*P* < 0.05 (D-G). “N. S.” denotes a lack of statistical difference (*P* > 0.05). The experiments depicted in this figure were replicated five times (*n* = 5, biological repeats), consistently yielding similar results. The scale bar corresponds to 100 μm.

The activation of Akt signaling cascade plays a crucial role in the initiation and progression of UVM [10, 11, 26]. Building upon our prior research that highlighted PCK1’s significance in activating Akt in endothelial cells [22], we undertook an analysis of PCK1’s impact on Akt activation in UVM cells. In 92.1 cells, the silencing of PCK1 using “PCK1-shRNA-s1” and “PCK1-shRNA-s2” (as shown in Figs. 2 and 3) led to a substantial inhibition of Akt Ser-473 phosphorylation (Fig. 6A). Total expression of Akt1 remained unchanged (Fig. 6A). Additionally, the utilization of CRISPR/Cas9 to KO PCK1 (as illustrated in Fig. 4) resulted in a similar outcome in 92.1 cells, where Akt activation was inhibited and Akt Ser-473 phosphorylation was downregulated (Fig. 6B). Total Akt1 was again unchanged (Fig. 6B). Conversely, in 92.1 cells overexpressing PCK1 (“oePCK1”, see Fig. 5), Akt Ser-473 phosphorylation was significantly enhanced (Fig. 6C), with total Akt1 again unchanged (Fig. 6C). These findings collectively suggest that PCK1 plays a pivotal role in Akt activation in UVM cells.

To investigate the connection between PCK1-driven UVM cell growth and the activation of Akt, we introduced a constitutively-active mutant Akt1 (caAkt1) with the S473D mutation into PCK1-silenced 92.1 cells (expressing “PCK1-shRNA-s2”). Our findings revealed that caAkt1 fully restored Akt phosphorylation in PCK1-shRNA-s2-expressing 92.1 cells (Fig. 6D), while leaving PCK1 protein expression unaffected (Fig. 6D). Importantly, caAkt1 substantially counteracted the inhibitory effects of PCK1 silencing on cell proliferation (Fig. 6E), migration (Fig. 6F). It also mitigated apoptosis activation in PCK1-silenced cells (Fig. 6G). Thus, the primary mechanism potentially underlying PCK1-driven UVM cell growth involves the mediation of Akt cascade activation

### PCK1 regulates Gαi3 expression in uveal melanoma cells

**Fig. 7:**
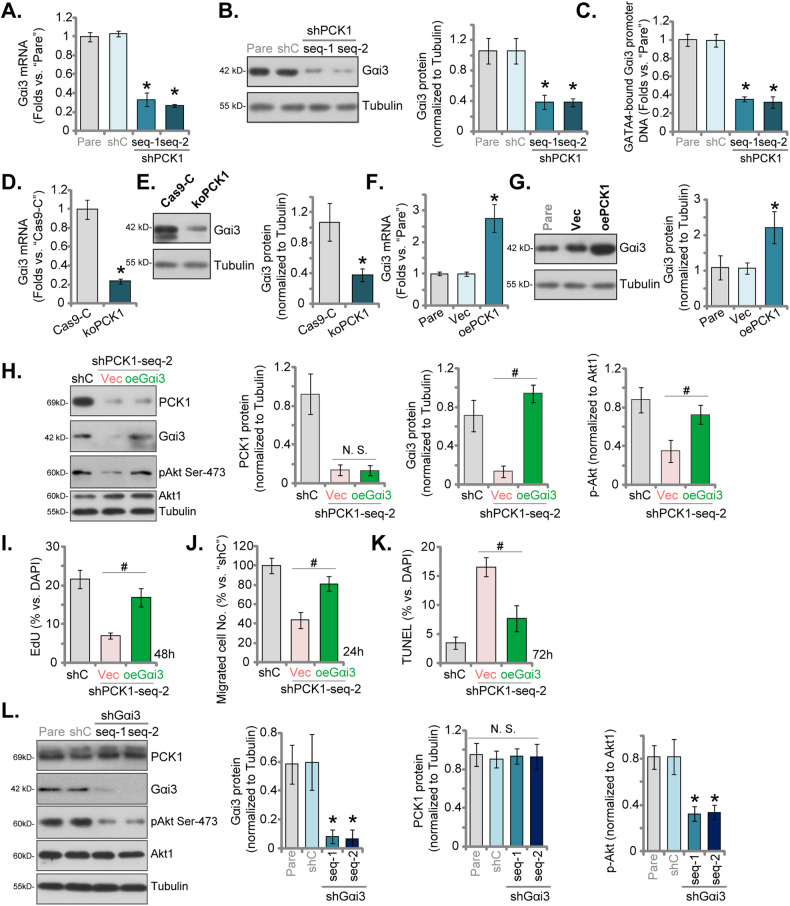
PCK1 regulates Gαi3 expression in uveal melanoma cells. The 92.1 cells were engineered to stably express the lentiviral PCK1 shRNA (“shPCK1-seq-1” or “shPCK1-seq-2”, the lentiviral control shRNA (“shC”) (**A**–**C**), the lentiviral CRISPR/Cas9-PCK1-KO construct (“koPCK1”), the CRISPR/Cas9-control construct (“Cas9-C”) (**D**, **E**), the lentivirus-packed PCK1-expressing construct (“oePCK1”) or the empty vector (“Vec”) (**F**, **G**), the mRNA and protein expression of Gαi3 was tested (**A**, **B**, **D–G**). ChIP assay showed the relative levels of *G*α*i3* promoter DNA binding to GATA4 in shC cells and PCK1-silenced cells (**C**). The shPCK1-seq-2-expressing 92.1 cells were subjected to stable transduction with the lentivirus-packed Gαi3-expressing construct (“oeGαi3”) or an empty vector (“Vec”). The expression levels of the listed proteins were shown (**H**). An equal number of the aforementioned 92.1 cells were cultured for specific durations, cell proliferation, migration, and apoptosis were assessed using EdU-nuclei staining (**I**), “Transwell” assays (**J**), and TUNEL-nuclei staining (**K**), respectively, with results quantified. The stable 92.1 UVM cells with the lentiviral Gαi3 shRNA (“shGαi3-seq-1” or “shGαi3-seq-2”, two different sequences) or the lentiviral control shRNA (“shC”) were established, and expression of listed proteins was shown (**L**). The numerical values are presented as the mean ± standard deviation (SD). “Pare” signifies the parental control cells. *Indicates statistical significance (*P* < 0.05) when compared to “shC”/”Cas9-C”/”Vec” cells (**A**–**G** and **L**). ^#^*P* < 0.05 (H-K). “N. S.” denotes a lack of statistical difference (*P* > 0.05). The experiments depicted in this figure were replicated five times (*n* = 5, biological repeats), consistently yielding similar results.

In our prior research, we demonstrated the significance of the interaction between PCK1 and the transcription factor GATA binding protein 4 (GATA4) in facilitating the serine phosphorylation of GATA4 [22]. This phosphorylation event, in turn, plays a crucial role in enhancing the transcription and expression of *Gαi3*, leading to an augmented activation of Akt in endothelial cells [22]. Consequently, we investigated the role of PCK1 in regulating Gαi3 expression in UVM cells. In 92.1 UVM cells, the silencing of PCK1 using “shPCK1-seq-1” or “shPCK1-seq-2” resulted in a significant decrease in both *Gαi3* mRNA (Fig. 7A) and protein levels (Fig. 7B). Furthermore, the knockdown of PCK1 led to a reduction in GATA4 binding to the *Gαi3* promoter DNA in 92.1 cells (Fig. 7C). Additionally, CRISPR/Cas9-induced PCK1 KO also led to a decrease in *Gαi3* mRNA (Fig. 7D) and protein (Fig. 7E) in 92.1 cells. Conversely, the overexpression of PCK1 (“oePCK1”) in 92.1 cells resulted in an upregulation of *Gαi3* mRNA (Fig. 7F) and protein (Fig. 7G). These findings support the importance of PCK1 in regulating Gαi3 expression in UVM cells. Next, the lentivirus-packed Gαi3-expressing construct (“oeGαi3”) was stably transduced to PCK1-shRNA-s2-expressing 92.1 cells. As shown, oeGαi3 restored Gαi3 expression and Akt activation, without affecting PCK1 expression in 92.1 cells (Fig. 7H). Importantly, the proliferation inhibition induced by PCK1 silencing (Fig. 7I), the inhibition of migration (Fig. 7J), and the induction of apoptosis (Fig. 7K) were alleviated by oeGαi3 in 92.1 cells. Subsequent investigations demonstrated that the suppression of Gαi3, achieved through the application of two distinct lentivirus-delivered shRNAs (“shGαi3-seq-1” and “shGαi3-seq-2”), resulted in the inhibition of Akt phosphorylation in 92.1 UVM cells (Fig. 7L), and PCK1 expression was unchanged (Fig. 7L). These findings collectively suggest that PCK1-mediated Akt activation is possibly attributed to the promotion of Gαi3 expression.

### PCK1 silencing inhibits UVM cell growth in vivo

**Fig. 8:**
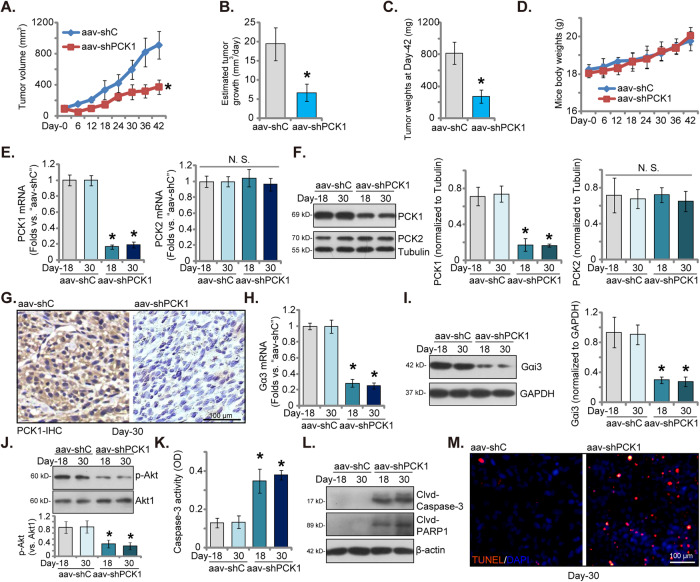
PCK1 silencing inhibits UVM cell growth in vivo. Xenograft-bearing nude mice with priUVM tumors were treated with intratumoral injections of adeno-associated virus (aav) carrying PCK1 shRNA (“aav-shPCK1”) or the control aav carrying scramble shRNA (“aav-shC”); Tumor volumes (in mm^3^, **A**) and the weights of the animals (in grams, **D**) were measured every six days. The daily priUVM xenograft growth rate (in mm^3^ per day) was also calculated (**B**). At Day 42, all priUVM xenografts were surgically removed and weighed (**C**). The expression of listed mRNAs and proteins in the described priUVM xenograft tissues was analyzed (**E**, **F**, **H**, **I**, **J** and **L**), with the Caspase-3 activity in tissue lysates assessed (**K**). Additionally, priUVM xenograft sections were subjected to immunohistochemistry (IHC) to detect PCK1 (**G**), or immunofluorescence to detect TUNEL-positive nuclei (**M**). Data are presented as mean ± standard deviation (SD). In **A**–**D**, each experimental group consisted of ten mice (*n* = 10); In **E**–**M**, five random tissue pieces within each male xenograft were measured (*n* = 5). *Indicates statistical significance (*P* < 0.05) when compared to “aav-shC” group. “N. S.” denotes a lack of statistical difference (*P* > 0.05). The scale bar corresponds to 100 μm.

Finally, we investigated the potential impact of PCK1 on UVM cell growth in vivo. The priUVM primary cells, in a quantity of 6 × 10^6^ cells per mouse, were subcutaneously injected into the flanks of nude mice. The subcutaneous priUVM xenografts were formed 21 days after cell injection, marked as “Day-0”. Subsequently, the nude mice carrying priUVM xenografts were randomly divided into two groups, each consisting of 10 mice (*n* = 10). One group received adeno-associated virus (aav) carrying PCK1 shRNA (“aav-shPCK1”), while the other group received a control aav carrying scramble shRNA (“aav-shC”). The virus was directly injected into the priUVM xenografts, and this injection process was repeated twice with a 48-hour interval between injections. Tumor volumes were recorded every six days. The results revealed a remarkable hindrance in priUVM xenograft growth after aav-shPCK1 injection, with significantly smaller volumes observed in the aav-shPCK1-treated xenografts compared to those in the control group (Fig. 8A). Moreover, the daily growth of priUVM xenografts, measured in mm^3^ per day, was notably reduced following aav-shPCK1 injection (Fig. 8B). On “Day-42”, or 42 days after the initial virus injection, all priUVM xenografts from both groups were harvested and individually weighed. The priUVM xenografts in the aav-shPCK1 group were significantly smaller and lighter than those in the control group (Fig. 8C). There was no significant difference in the weights of the nude mice between the two groups (Fig. 8D).

On the 18th day (“Day-18”) and 30th day (“Day-30”) following the initial virus injection, one priUVM xenograft from each group was isolated, and four priUVM xenografts obtained. A portion of these xenografts was then processed and homogenized for analysis. The results unveiled a substantial reduction in both *PCK1* mRNA (Fig. 8E) and protein (Fig. 8F) expression in the priUVM xenograft tissues injected with the PCK1-shRNA virus. The mRNA and protein expression of PCK2 remained unchanged (Fig. 8E, F). Immunohistochemistry (IHC) staining further affirmed the effective downregulation of PCK1 in the xenografts treated with aav-shPCK1 (Fig. 8G). Additionally, the mRNA and protein expression of Gαi3 was decreased in the PCK1-silenced priUVM xenograft tissues (Fig. 8H, I). Furthermore, p-Akt was reduced in priUVM xenograft tissues with PCK1 silencing (Fig. 8J).

Upon further investigation of the xenograft tissues, we observed an increase in Caspase-3 activity in the priUVM xenograft tissues injected with aav-shPCK1 (Fig. 8K). This increase was associated with elevated levels of cleaved-Caspase-3 and cleaved-PARP1 (Fig. 8L), indicating the activation of apoptosis. Moreover, immunofluorescence results from tissue slides provided additional evidence of apoptosis activation in PCK1-silenced priUVM xenografts, as evidenced by the increased percentage of TUNEL-positive apoptotic nuclei (Fig. 8M). In summary, these findings collectively demonstrate that aav-shPCK1 injection leads to PCK1 silencing, downregulation of Gαi3, inhibition of the Akt pathway, and induction of apoptosis in priUVM xenografts.

## Discussion

Recent research has suggested a novel, non-gluconeogenic role for PCK1, including acting as a protein kinase [27]. This newfound function has been shown to stimulate the growth, migration, and metastasis of various human cancer cells [13, 27–30]. In hepatocellular carcinoma (HCC) cells, the inhibition of PCK1 reduced the phosphorylation of INSIG (insulin-induced gene) 1/2, leading to a suppression of cell proliferation and a decrease in tumorigenesis [27, 29]. Shao and colleagues reported that PCK1 promoted the activation of nuclear SCAP-sterol regulatory element-binding protein 1 (SREBP1) in lung cancer cells, thereby contributing to cancer growth [28]. Furthermore, under hypoxic conditions, PCK1 has been shown to support pyrimidine nucleotide biosynthesis, which is crucial for the metastatic growth of colorectal cancer [30]. In the study by Zhu et al., it was revealed that overexpression of PCK1 is involved in promoting the growth of pancreatic cancer cells by mediating Akt activation [31].

**Fig. 9:**
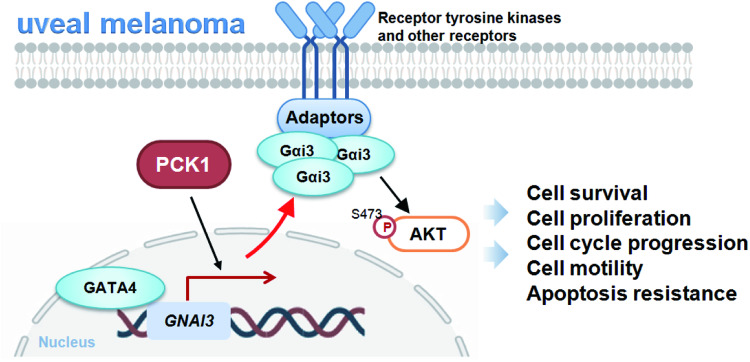
The proposed signaling cascade carton of the study. The results suggest that PCK1 promotes Gαi3 expression possibly by enhancing the transcriptional activity of GATA4. This, in turn, leads to increased Akt activation, thereby facilitating the growth of UVM cells both in vitro and in vivo. Considering the established role of PCK1 in angiogenesis, which is pivotal for UVM progression, these findings underscore PCK1 significant role in advancing UVM progression.

The findings of the present study hint at the possibility that PCK1 could be considered as an important tumor-promoting protein and a potential therapeutic target of UVM. PCK1 expression is upregulated in various UVM tissues as well as in primary UVM cells and immortalized lines. Moreover, bioinformatic studies revealed a correlation between PCK1 overexpression in UVM and advanced disease stages, along with reduced patient survival. In both primary and immortalized UVM cells, silencing PCK1 using viral shRNA or knocking it out via CRISPR/Cas9 substantially reduced cell viability, proliferation, cell cycle progression, and mobility, while inducing apoptosis. Conversely, upregulating PCK1 via a viral construct enhanced UVM cell proliferation and migration. In the in vivo setting, intratumoral injection of PCK1 shRNA aav significantly hindered the growth of subcutaneous xenografts formed by primary human UVM cells. These findings suggest a potential significance in the increased expression of PCK1 in the growth and progression of UVM cells (see proposed signaling carton in Fig. 9).

The hyperactivation of Akt in UVM results from a complex interplay of genetic modifications, encompassing mutations in upstream signaling molecules including receptor tyrosine kinases (RTKs) and the loss of tumor suppressor genes such as *PTEN* [10, 11, 26, 32–34]. Furthermore, disturbances in G protein-coupled receptor (GPCR) signaling, intricate feedback mechanisms, and cross-talk with other signaling pathways collectively contribute to the sustained activation of Akt [10, 11, 26, 32–34]. This multifaceted hyperactivation of Akt plays a pivotal role in promoting the survival, proliferation, and growth of UVM cells [10, 11, 26, 32–34]. Investigating the precise underlying mechanisms of Akt hyperactivation in UVM remains an active area of research, with significant implications for the development of targeted therapies.

The findings from this study suggest a potential role for PCK1 in driving Akt activation within UVM cells. We observed a decrease in Akt phosphorylation upon PCK1 silencing or knockout, while conversely, Akt phosphorylation increased with PCK1 overexpression in UVM cells. Importantly, the reestablishment of Akt phosphorylation through a constitutively active mutant Akt1 (S473D) mitigated the growth inhibition, migration suppression, and apoptosis triggered by PCK1 silencing in UVM cells. Additionally, a marked reduction in Akt activation was evident in PCK1-silenced UVM xenografts. Therefore, it’s possible that facilitating Akt activation could represent a potential mechanism underlying PCK1-driven growth of UVM cells (Fig. 9).

In our mechanistic investigation, we hypothesize that the PCK1-promoted Akt activation may potentially result from the upregulation of Gαi3 in UVM cells. The Gαi family of proteins consists of three members: Gαi1, Gαi2, and Gαi3 [35–37]. These proteins interact with ligand-activated GPCRs. A key role of Gαi proteins is to suppress adenylyl cyclase activity, resulting in a reduction of intracellular cyclic AMP (cAMP) levels [35–37]. Additionally research, including our own studies [38–41], has revealed Gαi proteins, particularly Gαi1 and Gαi3, play unconventional roles in signaling for non-GPCR receptors [38–46]. Gαi proteins can form associations with ligand-activated RTKs, such as EGFR (epidermal growth factor receptor) [47], keratinocyte growth factor receptor (KGFR) [38], VEGFR2 (vascular endothelial growth factor receptor 2) [41], BDNF receptor TrkB [46] and SCF (stem cell factor) receptor c-Kit [43], resulting in the activation of downstream Akt-mTOR and Erk cascades. Additionally, non-RTK receptors, including the Netrin-1 receptor CD146, R-spondin3 (RSPO3) receptor LGR4 (leucine-rich repeat-containing G Protein-coupled receptor 4) [42], IL-4 receptor IL-4R [40], and lipopolysaccharide (LPS) receptor TLR4 (Toll-like receptor 4) [48], also rely on Gαi1 and Gαi3 to transmit Akt-mTOR signaling.

In our recent study, it was shown that the interaction between PCK1 and GATA4 correlated with the phosphorylation of serine residues on GATA4, which in turn affect the transcription and expression of Gαi3, consequently affecting Akt activation in endothelial cells [22]. Here we suggest a potential involvement of PCK1 in the regulation of Gαi3 expression within UVM cells. Upon PCK1 silencing or knockout in UVM cells, there was an observed decrease in both mRNA and protein levels of Gαi3, whereas overexpression of PCK1 led to an increase in these levels. Moreover, PCK1 silencing inhibited GATA4-mediated Gαi3 transcription in UVM cells. Additionally, a reduction in Gαi3 expression was evident in UVM xenograft tissues following PCK1 silencing. Interestingly, the introduction of exogenous Gαi3 restored Akt activation and mitigate the suppressive effects observed in UVM cells due to PCK1 silencing. Hence, it is possible that PCK1 stimulates Gαi3 expression, causing heightened Akt activation to facilitate UVM cell growth both in vitro and in vivo. (Fig. 9).

## Materials and methods

### Reagents and chemicals

All viral constructs were previously detailed in our earlier publication [22]. List of the antibodies with relative concentration used were shown in Table 1. Polybrene, puromycin, caspase inhibitors, antibiotics, serum, and medium were obtained from Sigma (Shanghai, China). The remaining chemicals and fluorescence dyes were specified in our prior study [22].

**Table 1:** List of the antibodies with relative concentration used.

Antibodies	Company	Catalog number	Concentration
Rabbit polyclonal anti-PCK1	Boster Biological Technology	A02022-3	WB 1:1000 IHC 1:100
Rabbit polyclonal anti-PCK2	Cell Signaling Technology	6924	1:1000
Rabbit polyclonal anti-Cleaved Caspase-3	Cell Signaling Technology	9661	1:1000
Rabbit polyclonal anti-Cleaved Caspase-9	Cell Signaling Technology	9501	1:1000
Rabbit monoclonal anti-Cleaved PARP	Cell Signaling Technology	5625	1:1000
Rabbit monoclonal anti-phospho-Akt (Ser473)	Cell Signaling Technology	4060	1:2000
Mouse monoclonal anti-Akt1	Santa Cruz Biotechnology	sc-5298	1:2000
Mouse monoclonal anti-Gαi3	Santa Cruz Biotechnology	sc-365422	1:2000
Mouse monoclonal anti-GAPDH	Proteintech	60004-1-Ig	1:10000
Mouse monoclonal anti-β-actin	Proteintech	66009-1-Ig	1:10000

### Cells

The establishment and culture of primary human melanocytes were reported in our previous study [38]. The immortalized UVM cell lines, including 92.1, OCM-1 and Mel202, were provided by the Cell Bank of Shanghai Institute of Biochemistry and Cell Biology (Shanghai, China). Cells were maintained under DMEM/RMPI medium containing serum and antibiotics. For culturing primary UVM cells, the fresh UVM tissues, from one written-informed consent patient, were cut into small pieces and were subjected to enzymatic digestion using a 0.25% trypsin-EDTA solution for 20 minutes. Cells were subsequently cultured as monolayers at 37 °C in a humidified environment composed of 95% air and 5% CO_2_, using MEM medium supplemented with 10% serum. The fibroblasts, vessel cells and immune cells were abandoned. Subconfluent cell monolayers were trypsinized with a 0.25% trypsin-EDTA solution and were then seeded in a tissue culture dish at a density of 5 × 10^4^ cells/cm^2^, and the culture medium was refreshed every 2–3 days. Cells were all under examination to detect mycoplasma and microbial contamination. Verification of cell genotypes was carried out through various assessments, including STR profiling, population doubling time analysis, assessment of cell morphology. The protocols were approved by the Ethics Committee of Nanjing Medical University, in accordance with Declaration of Helsinki.

### Human tissues

UVM tissues, referred to as “T” tissues, and their corresponding normal tissues from the surrounding areas, designated as “N” tissues, were collected during surgical procedures. These samples were obtained from a cohort of nine primary UVM patients treated with surgical tumor resection surgeries at our institution. In compliance with the principles of the Declaration of Helsinki, written-informed consent was acquired from each tissue donor. The handling of human tissues strictly adhered to ethical guidelines and received approval from the Ethics Board of Nanjing Medical University.

### Immunohistochemistry (IHC)

The paraffin-embedded xenograft sections were subjected to baking, dewaxing, and hydration. Next, the tissue sections were washed with a 0.4% Triton X-100 in PBS (PBST) solution, and to mitigate non-specific binding they were incubated with 7.5% serum in PBS-T for 20 minutes. Subsequently, endogenous peroxidase activity was blocked by using hydrogen peroxide (H_2_O_2_), and the primary antibody (anti-PCK1) was applied with an incubation period of 12 h. A biotin-labeled IgG antibody was then applied for 2 h, followed by an incubation with streptavidin-HRP. Finally, diaminobenzidine (DAB) staining was utilized for visualization.

### Tissue fluorescence staining

The paraffin-embedded tissue slides underwent similar steps of baking, dewaxing, and hydration. Subsequently, tissue slides were washed with PBST solution and blocked with goat serum. TUNEL tissue kit was then utilized and the stained tissue sections were then examined using a confocal microscope (Zeiss).

### shRNA

Two distinct lentivirus-packed shRNAs in the GV248 vector (hU6-MCS-CBh-IRES-puromycin), each targeting unique, non-overlapping sequences of *PCK1*, were reported in our previous study and were designated as “shPCK1-seq-1” and “shPCK1-seq-2” [22]. Lentivirus was generated by transducing these constructs along with lentiviral packaging constructs into HEK-293 cells. UVM cells were seeded in six-well plates, reaching 50-60% confluence, and cultivated in a complete medium containing polybrene. Subsequently, they were infected with the lentivirus at a multiplicity of infection (MOI) of 10. After a 36 h incubation, cells were exposed to puromycin (3.0 μg/mL)-enriched complete medium for an additional 4-5 passages. The efficacy of PCK1 silencing in these stable cells was confirmed through Western blotting and qRT-PCR assays. As a control, lentivirus containing a scramble non-sense shRNA (“shC”) was added to the control cells [22]. The two lentivirus-packed Gαi3 shRNAs, “shGαi3-seq-1” and “shGαi3-seq-2”, were provided by Dr. Cao [49]. For in vivo studies, the shPCK1-seq-2 sequence was inserted into an adeno-associated virus (aav) construct from Dr. Yin [50]. The construct, along with the aav helper plasmids (Genechem, Shanghai, China), were co-transfected to HEK-293 cells via Lipofectamine 3000. After 48 h, the AAV particles were then harvested from the transfected cells and culture supernatant, which were then purified through ultracentrifugation and their titer was quantified using quantitative PCR (qPCR).

### CRISPR/Cas9-mediated PCK1 knockout (KO)

To achieve PCK1 knockout (KO), we initially transduced UVM cells with the Lenti-Cas9-puro construct [22]. Stable cell lines were established following puromycin selection. Subsequently, we introduced a lentiviral CRISPR/Cas9-PCK1-KO construct (containing sgRNA targeting human *PCK1*, as reported early [22, 31]), into the Cas9-expressing UVM cells. Stable colonies were again established after puromycin selection. These cells were then distributed into 96-well plates for screening to confirm the successful KO of PCK1. Ultimately, a single stable cell line with *PCK1* KO, referred to as “koPCK1”, was created. Control Cas9-expressing UVM cells were stably transduced with a lentiviral CRISPR/Cas9-construct with non-sense control sgRNA.

### Gene overexpression

To overexpress PCK1, UVM cells were initially seeded in six-well plates and were cultured in a complete medium containing polybrene. When cells reached 50-60% confluence, the lentivirus-packaged PCK1-overexpressing GV492 construct, also reported in our previous study [22], was introduced. Stable cells were generated after puromycin selection. The successful overexpression of PCK1 was confirmed through qRT-PCR and Western blotting assays. Control cells were stably transduced with the GV492 empty vector. The lentivirus-packed Gαi3-expressing construct (from Dr. Cao [49]) was utilized to establish Gαi3-overexpressing cells using the same procedure.

### CCK-8 (cell counting kit-8) assay

In the CCK-8 assay, UVM cells with the specified genetic modifications or treatments were seeded into 96-well plates at a density of 4,500 cells per well. After the described incubation period, 8 mg/mL of CCK-8 was added for an additional 2 h. The absorbance (optical density, OD) at 450 nm for each well was measured using a plate reader (Bio-Tek Instruments, Hopkinton, MA).

### EdU (5-ethynyl-2’-deoxyuridine) staining

For EdU staining, UVM cells were initially placed into 12-well plates at a density of 40,000 cells per well. After 48 h, the cells were fixed and permeabilized. Subsequently, EdU and DAPI (4’,6-diamidino-2-phenylindole) dyes were added, and the cells were observed under a fluorescence microscope (Zeiss).

### Transwell assays

In Transwell assays, UVM cells with the specified genetic modifications were cultured in serum-free medium and placed into the upper chambers of Transwell inserts (12,000 cells per chamber, Millipore, Billerica, MA). Complete medium containing serum was added to the lower chamber. The cells were allowed to migrate for 24 h. Migrated cells on the lower surface were fixed and stained with crystal violet. For in vitro invasion assays, the Transwell chambers were pre-coated with Matrigel (Sigma).

### Chromatin Immunoprecipitation (ChIP)

ChIP assay protocols were previously detailed [22]. In brief, cell lysates were sonicated using a Misonix Sonicator 3000 Homogenizer, resulting in fragmented genomic DNA. These lysates, diluted in ChIP dilution buffer, were subjected to immunoprecipitation with an anti-GATA4 antibody (Abcam). The DNA bound by GATA4 was eluted from protein A/G agarose, and NaCl was added to reverse cross-linking between proteins and genomic DNA. The DNA containing the proposed conserved *Gαi3* promoter site [51] was subsequently analyzed via quantitative PCR (qPCR).

### Constitutively-active mutant Akt1

As described, the recombinant lentivirus expressing the constitutively-active Akt1 (caAkt1, S473D) [52] was added to cultured UVM cells. Afterwards, cells were selected using puromycin. The expression of caAkt1 was validated through Western blotting assays.

### Other assays

Various additional assays were performed, including Western blotting, qRT-PCR, the use of JC-1 dye to assess mitochondrial depolarization, propidium iodide (PI)-flow cytometry for evaluating cell cycle progression, TUNEL (Terminal deoxynucleotidyl transferase dUTP Nick End Labeling) staining and Annexin V-PI FACS assay to measure cell apoptosis, Trypan blue staining to detect cell death, and the assessment of Caspase-3 activity and cytochrome C using ELISA assays. Detailed descriptions of these assays can be found in our previous studies [22, 53, 54]. JC-1 green fluorescence intensity was quantified via the Olympus CellSens software. The uncropped blotting images are in Figure S1. Quantification of microscopy results involves analyzing five distinct microscopic fields from each independently treated sample (five biological repeats).

### Animal studies

In xenograft experiments, four- to five-week-old nude mice, ensuring an even distribution of both genders, with an average weight ranging from 17.8 to 18.2 grams were utilized. These mice were housed at the animal facility of authors institutions Subcutaneous (*s.c*.) injections of six million UVM cells suspended in 0.2 mL of Matrigel-containing serum-free medium were administered to each mouse. This procedure resulted in the development of UVM xenografts within 21 days, with each reaching a volume close to 100 mm^3^. Subsequently, intratumoral injections of specified adeno-associated virus (aav) were carried out, delivering 0.8 μL of virus per xenograft, totaling 1.0 × 10^9^ PFU, and administered twice, with a 48-hour interval. Tumor dimensions were measured, and volumes were estimated using a previously established formula [39, 55, 56]. All animal procedures received ethical approval from the Institutional Animal Care and Use Committee (IACUC) and the Ethics Board of Nanjing Medical University.

### Statistical analysis

The numerical data, with normal distribution, were presented as mean ± standard deviation (SD). To assess variations among three or more groups, we performed a one-way analysis of variance (ANOVA), followed by Tukey’s multiple comparison test, utilizing GraphPad Prism software. When comparing two groups, the Student t-test was applied within Microsoft Excel. Statistical significance was defined as a *P*-value below 0.05.

### Supplementary information

Figure S1.

## Data Availability

All data generated during this study are included in this published article. Data will be made available upon request.
