# Supplementary material for: Expression and functional significance of phosphoenolpyruvate carboxykinase 1 in uveal melanoma
Source: Cell Death Discov. 2024 Apr 26;10:196. doi: 10.1038/s41420-024-01963-y (PMC11053060; doi:10.1038/s41420-024-01963-y)

Figure S1: The uncropped blotting images.

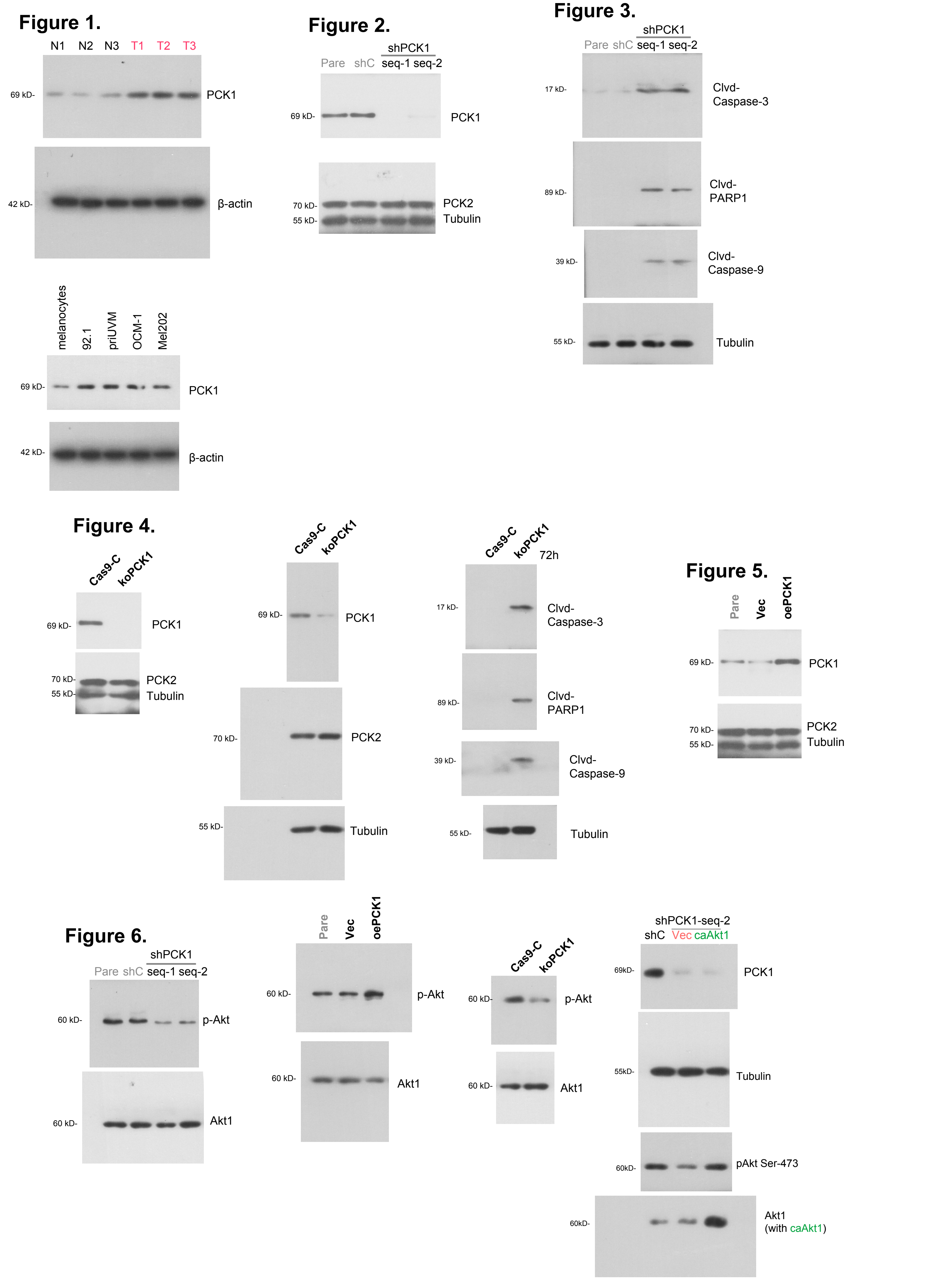

Figure 7.

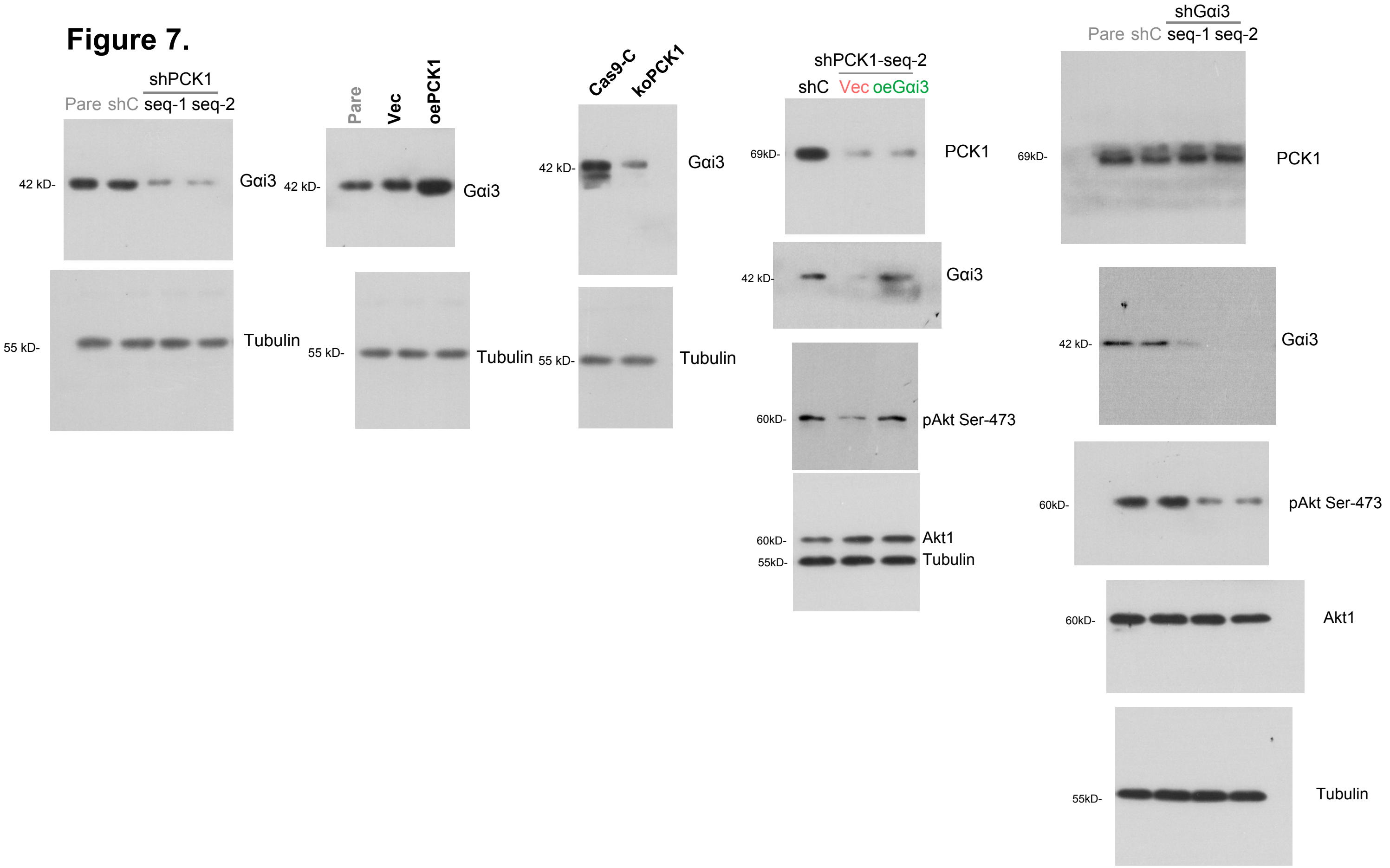

Figure 8.

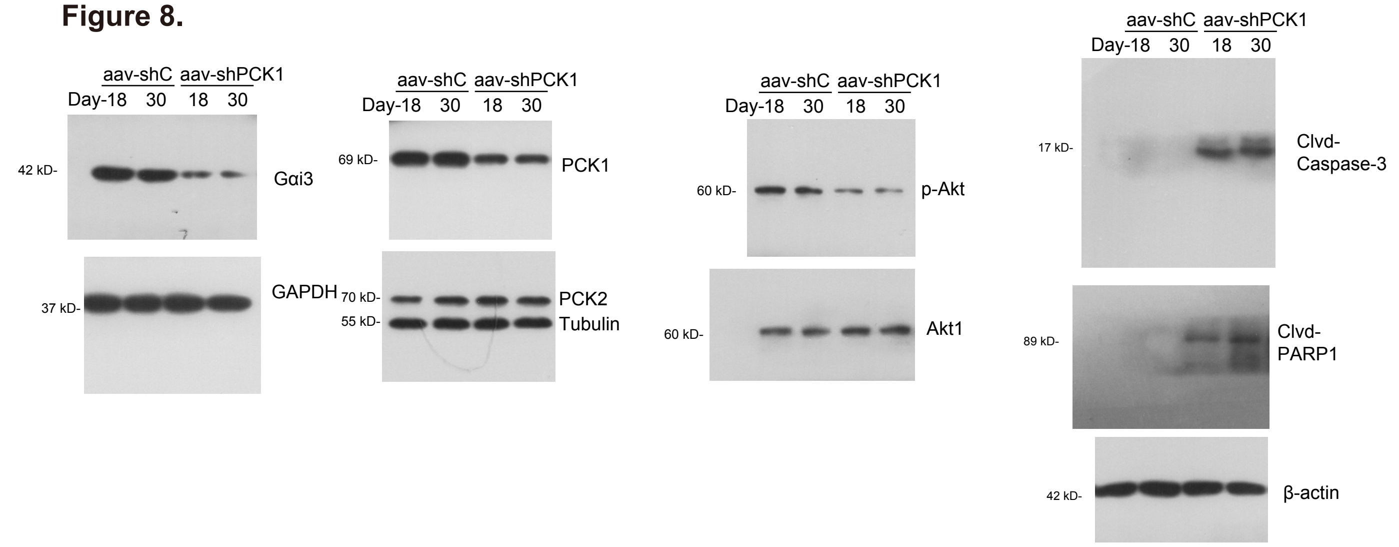

Supplement: Supplementary file 1 — Figure S1. [file 41420_2024_1963_MOESM1_ESM.pdf]
